# Palliative care in home health care services and hospitals – the role of the resource nurse, a qualitative study

**DOI:** 10.1186/s12904-022-00956-x

**Published:** 2022-05-03

**Authors:** Håkon Johansen, Vigdis Abrahamsen Grøndahl, Ann Karin Helgesen

**Affiliations:** Faculty of Health, Welfare and Organisation, Østfold University College, Halden, 1757 Norway

**Keywords:** Resource nurse, Palliative care, Hospitals, Home health care services

## Abstract

**Background:**

The role of the resource nurse aims at bridging the gap between the specialist nurses and the nurses who work in non-specialist wards. The role is established internationally and used in a wide area of clinical settings. The resource nurse is promoting evidence-based practice.

Patients with life limiting conditions including cancer and other chronic diseases will likely need palliative care during the trajectory of illness. Due to the complexity of palliative care, both interprofessional help and cooperation between levels of healthcare are considered necessary.

**Aim:**

The aim of this study was to explore the perceptions and experiences related to the role of the resource nurse in palliative care in the setting of home health care services and hospitals in Norway, from the perspectives of the resource nurses and the ward nurses.

**Design:**

The study has an explorative design with a qualitative approach.

**Methods:**

Eight individual interviews were conducted. Audiotaped interview material was transcribed verbatim and the data were analysed using systematic text condensation. The encoded data material provided the basis for writing analytical texts that in turn resulted in meaningful descriptions of the different categories.

**Results:**

Four resource nurses and four ward nurses participated in individual interviews.

Analysis of the data yielded three categories: 1. Expectations of better competence in the ward. 2. Expectations of better cooperation between professions and different levels of healthcare services. 3. Improvements and hindrances.

**Conclusion:**

The resource nurse role is underutilized due to heavy workload and inefficient organization of care. Improvements such as sufficient time resources, support from the ward nurse and cooperation with staff nurses, the resource nurses’ role could contribute to increased competence and cooperation interprofessionally and between levels of healthcare.

**Supplementary Information:**

The online version contains supplementary material available at 10.1186/s12904-022-00956-x.

## Background

The term resource nurse or the parallel term link nurse or nurse champion denotes a healthcare role that is established internationally and is used in a wide area of clinical settings such as infection prevention and control [1], wound and skin care [2], diabetes care [3] and palliative care [4, 5]. However, neither the resource nurses’ role nor the similar terms are distinctively defined [6, 7]. The role of the resource nurse and similar terms aim at bridging the gap between the specialist nurses and the nurses who work in non-specialist wards [7]. Furthermore, the resource nurse role aims at promoting evidence-based practice [6]. The resource nurse is a practising nurse with interest in a field of nursing speciality and who has ongoing contact with the specialist competence and an obligation to help enhance competence among their colleagues [8].

The Norwegian national strategy for palliative care [9, 10] recommends appointing a resource nurse at units (nursing homes, home health care services and hospitals) that have seriously ill and dying patients. The appointed nurse acts as a resource for the patient and next of kin. The resource nurse also works with system improvements related to routines, procedures and pathways, in addition to advising and guiding colleagues, disseminating new knowledge and initiating reflection [9].

Globally, the need for palliative care is increasing, but not everyone needing competent palliative care actually receives it [11]. Most patients with life limiting conditions including cancer and other chronic diseases will need palliative care during the trajectory of illness [12]. Palliative care aims to prevent and relieve suffering by early identification, assessment and treatment of pain and other problems. In addition, it aims to improve quality of life for both patients with life threatening illness and their families [13]. Most patients with life limiting conditions including cancer and other chronic diseases will experience physical, psychosocial and spiritual suffering and will need an interprofessional approach to alleviate suffering [12]. Interprofessional collaboration is considered necessary to address patient’s complex palliative care needs [14].

When the nurse is recognized as a partner in the collaboration with the physician and not merely as an assistant receiving orders, he or she can contribute significantly to patient outcomes [15].

Palliative care is mainly offered in settings where palliative care is not the main activity [4]. The major barrier to best evidence-based palliative care is limited training in palliative care along with a lack of awareness concerning what palliative care is and it’s importance for patients [11, 16]. Furthermore, many patients with life limiting conditions including cancer and other chronic diseases will need palliative care at home as well as in hospital throughout the illness trajectory. Hence, good quality of care presupposes cooperation between the different levels of healthcare [17].

Norway is ranked in the third quartile among developed countries in the Quality of Death Index overall score [18]. Quality of palliative care is considered to be generally good, but somewhat variable. Both healthcare personnel and patients report a need to enhance healthcare personnel’s competence [19, 20]. Internationally, research on the role of the resource nurse in palliative care in the context of home health care services (i.e. health services to persons living in their own home) and hospitals is limited, and such research is scarce in Norway. Further research might provide new knowledge that might contribute towards better understanding and potentially better utilization of those having the role of resource nurse in palliative care. Therefore, the aim of this study was to explore the perceptions and experiences related to the role of the resource nurse in palliative care in the setting of home health care services and hospitals in Norway, from the perspectives of the resource nurses and the ward nurses.

## Methods

### Design

The study has an explorative design with a qualitative approach as research is scant on the subject of the resource nurses’ role in palliative care in the context of home health care services and hospitals [21]. Semi structured, individual interviews were used to collect data. The sampling of informants was purposeful and consisted of resource nurses and the ward nurses in charge of each ward where the resource nurse worked. Four resource nurses and four ward nurses were invited to participate in the study. The study involved one home health care service and one hospital.

### Data collection

The interviews were conducted by the first author using an interview guide containing questions that were directed to the resource nurses and the ward nurses respectively. The interview guide was developed in discussion between the authors. The authors have during the last years conducted research on the theme “resource nurse” and the first author coordinates a network for resource nurses. Besides, scientific literature informed the interview guide. The resource nurses were asked about how they perceived their role, what worked well, what challenges they were confronted with, how they contributed to interprofessional collaboration and what they needed to do a good job. The ward nurses were asked about how they perceived having an appointed resource nurse, how the resource nurse contributed and what could be challenging. The data were collected at the home health care service and the hospital. No one else than participants and the researcher were present during the interviews. The interviews lasted from 30 to 60 min.

### Participants and setting

Resource nurse-participants were recruited by the ward nurse. The ward nurse-participants at the hospital were recruited by the head nurse at the cancer ward. The ward nurse-participant at the home health care service were recruited by the cancer coordinator in the municipality. The participating resource nurses were all female. Two of them worked in a home health care service department and two worked in a hospital. They had between five and 18 years of experience as registered nurses and between five months and three years’ experience as resource nurses. The participating ward nurses were all female and had between two and 13 years of experience as ward nurses. Both the home health care service department and the hospital were located in South-East Norway.

### Data analysis

The first author transcribed the interviews. After transcribing the audiotaped interview material verbatim, the data were analysed using systematic text condensation [22]. Phenomenology underpinned the systematic text condensation according to Malterud [22]. Our encoded data material provided the basis for writing analytical texts that in turn resulted in meaningful descriptions of the different categories. Themes were derived from the data. The first author conducted the analysis under supervision of the third author.

Firstly, the transcribed text was read several times, and presumptions were tentatively set aside. Six provisional themes emerged from the data material. Secondly, the text elements were examined thoroughly for elements which could help to answer the research question. The six provisional themes were refined into three categories, and the meaning units were coded within these three categories. Clearly distinctive phenomena were categorized in different codes, and facets of the same phenomenon were categorized in the same code. Resemblances and differences across and within the categories were reflected on. When meaning units did not fit into the three categories, we asked questions and made decisions about changing categorization throughout the analysis. Thirdly, the text was decontextualized, and we wrote new texts with text elements that belonged to the same category i.e., text condensation. Fourthly, the text was recontextualized into a comprehensive text that reflected the meanings of the participants. This was done by writing an analytical text based on the text condensations. To be sure that the analytical text reflected the meanings of the participants, the original transcription was examined looking for meaning units that could challenge the analytical text. This last step of the analysis resulted in some minor adjustments of the analytical text. The analytical text, along with illustrating quotations, will be presented in the results.

## Results

Four resource nurses and four ward nurses participated in individual interviews. Analysis of the data yielded three categories:

1. Expectations of better competence in the ward
2. Expectations of better cooperation between professions and different levels of healthcare services
3. Improvements and hindrances

### Expectations of better competence in the ward

The data shed light on an expectation from the ward nurses regarding better competence in the ward, and the resource nurses want to contribute.

The data showed that the ward nurses did not have sufficient time to maintain the level of competence in the ward by themselves. They explained that they wished to highlight the resource nurse’s role and make use of her potential, organize the resource nurse’s work to include planned days in the staff shift schedule and develop the role towards education, counselling, reflection and quality improvement.

*As a leader I do not have a chance to observe the quality and level of knowledge in the ward, so having a person that has the primary responsibility for that follows up and has the time for it. We could not have managed without*.

The ward nurses stated that patient safety is at risk when the level of competence in the ward is not satisfying. They described the necessity of professional personnel to provide safe and secure services. Furthermore, they stated that in a ward in continual development and frequently new personnel in need of training, the resource nurse was essential. Unfortunately, due to time constraints, the resource nurse’s role was not utilized to its full potential. This hindered professional development and caused personnel to feel insecure as well as necessary training to be halfway done, according to the ward nurses.

Another issue which described by the ward nurses was that the resource nurse had to take care of her own patients first, and secondly supervise her colleagues in order to keep them up to date and capable of dealing with challenging tasks.

*The threat is that the expectations are a bit too extensive because (being a resource nurse) is a duty in addition to the ordinary tasks (service to the patients).……. I don’t have the opportunity to just give her more time, because then I will not be able to solve the (ordinary) tasks.*

The ward nurses also mentioned the necessity of describing their expectations about the role of the resource nurses to them. They expected the resource nurse to show her competence and become confident in her role, and to be available for her colleagues.

*I support her to take her stand; you must take, you cannot wait to get. Show your competence. If your colleagues don’t listen, it is their problem, but you make yourself available.*

The ward nurses mentioned that the resource nurse met the patient in the early stages of care, often together with the specialist cancer care nurse in the municipality. This way she got a general view of the patients in need of cancer and palliative care and was thus prepared to inform and counsel her colleagues.

The resource nurses stated that they wish to convey new knowledge to their colleagues and keep them up to date, promote vocational and ethical reflection, and discuss proper care of the patients. The resource nurses said that they tried to answer questions from their colleagues and to inform them when there are new procedures, treatments, or medications to be updated.

*If we drive together, then maybe we can converse in the car, reflect and talk together; (we talk about) how we handled this or that situation, if it was good or if it was bad. You get better then, and maybe do a better job next time.*

According to the data, the ward nurses wanted to utilize bedside counselling as a tool to increase and maintain competence for the personnel. The resource nurse suggested that she might observe the caregiving of the novice colleague and counsel her afterwards, or to counsel through a show-and-tell in the actual situation.

### Expectations of better cooperation between professions and different levels of healthcare services

The data made clear that the ward nurse anticipated better cooperation and that the resource nurse had the potential to fulfil this expectation.

The data, both form the ward nurses and the resource nurses showed that every nurse in the ward participated in interdisciplinary cooperation regarding their own patients. This could result in patients not getting proper help, according to the resource nurses. They said that as resource nurses they could have contributed with their competence and experience in such situations.

The ward nurses described that they wanted the resource nurse to contribute to interdisciplinary cooperation and take responsibility for promoting the nurse’s perspective, e.g., make suggestions founded on the nurse’s perspective when an individual plan for a patient is made. In palliative care, interdisciplinary cooperation may sometimes be insufficient, but the resource nurse, with her competence, could help to remedy this, the ward nurses explained. The resource nurses said that they wanted to discuss matters with the physician and reach a common understanding of the different professional perspectives in decision making.

*I think we (the resource nurses) could participate and contribute; we are not really included there yet. That is the dream scenario; it is more every single nurse with her patients …. We (personnel on the ward) have the meeting before the doctor’s round twice a week with all the doctors and one nurse, but we (the resource nurses) do not participate there.*

The ward nurses said that there was no organized or planned interaction between different levels of healthcare services concerning the palliative care, such as face-to-face meetings, except for telephone calls and e-link when it was necessary. Sometimes the specialist cancer care nurse in the municipality invited the resource nurse to participate in meetings concerning a patient’ discharge. However, discharge meetings were rarely arranged, due to time constraints, the ward nurse informants said. The resource nurses highlighted the importance of meeting the physician and patient face to face before discharge. This provided an opportunity to understand what the patient wanted, and made it easier to know what to ask about.

Interaction between the levels of care was necessary e.g., when a patient needed equipment and assistance after discharge, when a patient’s situation at discharge was unclear and vague or when next of kin did not want the patient to come home. The resource nurses expressed that she would have been able to contribute in the interaction, but it is not organized that way.

*“for the time being I have not been involved in a situation (interaction with the municipality) …… but it would have been quite natural to assist, but this is not something that I can do here.*

### Improvements and hindrances

The data shed light on the fact that the resource nurse’s role is affected by circumstances that can further or hinder it.

The resource nurses described that they needed support and feedback from the ward nurse in order to fulfil the role as resource nurse.

*The leader must be interested in utilizing that resource; without embeddedness in leadership it will not be used…., the resource nurse must be engaged herself and the leader must be involved, too.*

The resource nurses described support from the ward nurse regarding the organization of the care and supportive feedback, but the resource nurses also noted that they did not get very much recognition or appreciation.

Busy days could impede reflection and competence building. The resource nurses said that they barely had time to sit down for lunch, and that the one-on-one reflections were done in a hurry.

The data also showed that it takes time before the resource nurse became confident and posed questions about the patients to her colleagues. Furthermore, the colleagues needed to feel confident in the cooperation with the resource nurse.

The ward nurses stated that the personnel were sometimes unwilling to listen to the resource nurse, because they were experienced nurses themselves and worked independently to a large extent.

*“When she said you must update yourself on the integrated care plan, then they did not care so much …. they know a lot and have a lot of experience; maybe they felt it was unnecessary …. but it is a good idea not to be too arrogant.”*

According to the resource nurses, it was important that the colleagues experienced that the resource nurse contributed positively, e.g. that she took care of issues or communicated issues to the ward nurse. Furthermore, the conversation had to be a dialogue where the resource nurse herself was open to criticism. If she is open, then it is possible to get approval of proposals that may not be popular.

*Even though I am the resource nurse, it is important that the other is receptive. Give the other nurse some space, then we can talk about this or that…*

According to the ward nurse informants, the resource nurse must enhance her own competence to be able to improve her colleague’s competence. Therefore, she needs time and access to newer knowledge, the resource nurse informants explained. The resource nurse was interested in a wide area of topics related to palliative care and read on the internet or consulted the nursing textbooks. Furthermore, by participating in the local network of resource nurses, which is facilitated by the specialist cancer care nurse, she acquired new knowledge, learned from peer resource nurses, and discussed current topics, according to the resource nurse informants.

*I seldom get a no if I ask to participate in (network-) meetings or courses, to increase my knowledge*

## Discussion

The aim of this study was to explore the perceptions and experiences related to the role of the resource nurse in palliative care in the setting of home health care services and hospitals in Norway, from the perspectives of the resource nurses and the ward nurses.

The data shed light on ward nurses’ expectations regarding better competence and better cooperation interprofessionally and between levels of healthcare; the resource role has the potential to fulfil these expectations. Moreover, the data illuminated the fact that the resource nurse’s role is affected by circumstances that can further or hinder it.

The results showed that the ward nurse had an expectation regarding better competence, but that the ward nurses did not have sufficient time to maintain the level of competence in the ward by themselves and wanted the resource nurse to take on competence maintenance and development. Moreover, the resource nurses want to contribute towards better care provision. Nevalainen et al. [23] reported that patient safety and quality of care require continuous professional development and maintenance of competence. A resource nurse might support nurses learning, make work-based learning possible and contribute to continuous professional development [23–25].

The results indicated that both the ward nurse, the resource nurse and the staff nurses had a high workload, and that this made competence maintenance difficult. Nevalainen et al. [23] reported that high workload reduce the possibility for staff members to interact and learn from each other. Furthermore, Nevalainen et al. [23] reported that though the ward nurse tend to undervalue the resource nurse influence, he or she can potentially play an important role in observing and improving competence. Other research underscored that competence improvement presupposes an organizational culture that recognizes continual professional development and allocates sufficient time and human resources [26]. Besides, experienced colleagues that provide support and act as role models are important for learning and patient outcomes, as Davis et al. [25] reported.

The results showed that an important means of competence development is bedside counselling. The resource nurse could stand beside the novice nurse and give support in the ongoing situation. Davis et al. [25] reported that nurses learn better when performing activities rather than watching the activity demonstrated. In this way the novice nurse gets feedback directly and in the situation from the more experienced colleague [23], and the novice can be challenged adequately [27]. Besides, learning activities closely related to practice enhance the nurse’s motivation to learn [28].

In contrast, the results indicated that heavy workload caused the resource nurse role to be underutilized. This is consistent with Engel et al. [4], who reported that trained nurse champions in palliative care are able and willing to contribute to a greater extent in competence activities.

The results indicated that personnel become insecure and are not well trained in a situation with limited professional development. A recent study [29] indicated that the resource nurse may contribute to make the unexperienced colleague more confident in performing palliative care.

Jantzen [24] reported that healthcare is characterized by change, and Davis et. al [25] reported that nurses who stopped learning were not able to adapt to continual development and new demands in medicine and nursing.

The results showed an expectation on the part of the ward nurse of better cooperation, but the organization of palliative care restricted the resource nurse’s opportunity to support cooperation. Every staff nurse, including the resource nurse herself, had to take care of their own patients. Consequently, this decreased the resource nurse’s scope of action and put up a barrier preventing her from supporting her colleagues in performing care and supporting cooperation interprofessionally and between levels of healthcare. Previous research has shown that availability of the experienced colleague for advice and support are a prerequisite for learning [25]. Furthermore, the results showed that this way of organizing reduced the resource nurse’s opportunity to care for the patients in most need of her competence. On the other hand, the results showed that the resource nurse got information about the patient in need of palliative care at an early stage of care and could use the knowledge she got about the patient in counselling her colleagues.

The results showed how the ward nurse communicated her expectations towards the resource nurse. Nevalainen et al. [23] reported that commitment to continuing development ought to be both confirmed and controlled. On the other hand, the results showed that the resource nurse needed support and feedback from the ward nurse, and that the ward nurse, through counselling, could help her to do her best. The results indicated a disparity regarding supportive feedback and recognition – some resource nurses seemed to get it, others seemed not to receive supportive feedback or recognition. A recent study [29] indicated that the ward nurse must appreciate the effort of the resource nurse, and that it is a prerequisite that the ward nurse acknowledges the importance of competence management. It seems reasonable to suggest that a close cooperation between the ward nurse and the resource nurse, along with supportive feedback, is necessary to succeed in competence management.

Moreover, the results showed that the resource nurse needs to be recognized by her colleagues as well. Sometimes the colleagues feel that they do not need counselling and/or new knowledge. This is consistent with Heals [7], who reported that health personnel may be reluctant to change established routines and ways of thinking and this can be a hindrance to new thinking and new practice and may perpetuate outdated practices [30]. Besides, according to Jantzen [24], an appreciative and supportive culture of the work community is a prerequisite for the resource nurse’s best performance. On the other hand, the results showed that the resource nurse needed to gain trust from her colleagues, via dialogue and by contributing positively. In the context of nursing students’ practical learning, Nielsen et al. [27] reported that it is vital that the preceptor is competent. Incompetence or uncertainty in clinical situations weakens the preceptee’s confidence in the preceptor. If the relationship between preceptee and preceptor is one of confidence, it is easier for the preceptee to admit a need for further training or knowledge [27]. According to Davis et al. [25] it is also necessary to acknowledge the colleague’s competence before conveying new knowledge. Therefore, it may seem reasonable to suggest that receptiveness and confidence are prerequisites for the collaboration between the resource nurse and her colleagues.

The results indicated that interprofessional cooperation is important for quality of palliative care. Earlier studies [14, 29, 31] reported that interprofessional cooperation is important for quality of palliative care. The results showed that the nurse’s perspective can contribute to better care, but that the resource nurse’s voice are not so often heard when the hospital doctors make plans for the patients. Champion-Smith et al. [32] reported that the doctors could be helped to think beyond the medical model in dialogue with nurses. This implies that the focus in an interprofessional meeting may be solely on physical care and hence compromising holistic care [33]. It is reasonable to suggest that the resource nurse with her experience and competence is the most capable of promoting the nurse’s perspective. Jantzen [24] reported that a change in organizational culture is necessary to recognize and value the experienced nurses. And according to Mlambo et al. [28] the resource nurse might also contribute to professional development among her colleagues, and make them confident in professional cooperation and capable of challenging medical decisions . Thereby, the resource nurse is the foremost advocate for the nurse’s perspective but also enables her colleagues to speak up in interprofessional cooperation [28].

The results also indicated that interprofessional meetings face-to-face are rare due to organization of palliative care and time constraints, and this may result in inadequate understanding of the patient and result in not a fully proper care. This is consistent with Engel [4], who reported that home health care nurses’ collaboration with hospital staff regarding patients in need of palliative care is limited, and that organizing and financing do not support more collaboration. The importance of meetings face-to-face is underscored by Neergaard et al. [31] who reported that this facilitates interprofessional cooperation in palliative care.

According to the results, the study participants highlighted the importance of the resource nurse participating in discharge meetings face-to-face. According to Lyngstad et al. [34], the use of e-link enhances connections between discharge collaboration parties, but it has as a consequence fewer face-to-face meetings [35]. In the context of collaboration between home health care providers and General Practitioners, Melby and Hellesø [35] reported that face-to-face meetings are important for efficient collaboration.

The results showed that the resource nurse must enhance her own competence to be able to increase the competence of her colleagues. She does so by reading on the internet or reading her nursing school textbooks as well as by participating in the local network of resource nurses. The specialist cancer care nurse facilitates the local network; the results also showed that through her participation in the local network, she obtains new knowledge, learns from the other resource nurses and discuses current topics.

Kamal et al. reported [5] that in a care champion network, the generalist level in healthcare meets the specialist level, and the care champions become the link between the specialist level and the generalist level. Practice needs to be reflected upon and corrected if necessary [24]. It may seem reasonable to suggest that the resource nurse can contribute to keep her colleagues up-to-date and capable of meeting a continously changing healthcare system.

### Study strengths and limitations

The study has a limited number of participants. However, the last interview did not provide any new information. In addition, the perceptions and experiences related to the resource nurse’s role in palliative care in hospitals and home health care services are explored by two perspectives.We did this by interviewing both the resource nurse and the ward nurse. Furthermore, by exploring in two different contexts, the home health care service and the hospital, we achieved nuanced information, especially regarding the resource nurse role in cooperation between levels of healthcare.

## Conclusion

The resource nurse role is underutilized due to heavy workload and inefficient organization of care. With improvements such as sufficient time resources, support from the ward nurse and cooperation with staff nurses, the resource nurses’ role could contribute to increased competence and cooperation interprofessionally and between levels of healthcare.

## Supplementary Information

**Additional file 1.** Interview guide.

## Data Availability

The datasets generated and/or analysed during the current study are not publicly available out of consideration for the informants and confidentiality but are available from the corresponding author on reasonable request.

## Abbreviation

NSD: Norwegian Centre for Research Data
