# Supplementary material for: Palliative care in home health care services and hospitals – the role of the resource nurse, a qualitative study
Source: BMC Palliat Care. 2022 May 3;21:64. doi: 10.1186/s12904-022-00956-x (PMC9063046; doi:10.1186/s12904-022-00956-x)
Supplement: Supplementary file 1 — Additional file 1. Interview guide. [file 12904_2022_956_MOESM1_ESM.pdf]

Palliative care in home health care services and hospitals – the role of the resource nurse, a qualitative study. Johansen, H., Grøndahl, V.A., Helgesen, A.K.

Interview guide, resource nurse

Can you please tell about how you perceive your role as a resource nurse?
Can you please tell about what worked well and what challenges you are confronted with?
Can you please tell about how you contribute to interprofessional collaboration?
Can you please tell about what you need to do a good job?

Interview guide, ward nurse

Can you please tell about how you perceive having an appointed resource nurse?
Can you please tell about how the resource nurse contribute to better competence and better collaboration?
Can you please tell about what could be challenging having an appointed resource nurse?
